# Efficacy of *Salmonella enterica* phage SW03 and Escherichia coli O157:H7 phage CF03 in food matrices under storage conditions

**DOI:** 10.3389/fmicb.2026.1858265

**Published:** 2026-06-19

**Authors:** Ohood Sallam, Mohamed T. Shaaban, Tareq M. Osaili, Amany A. M. Ahmed, Eman Fayad, Hana Alsufyani, Maha Alsunbul, Khalid M. Alsyaad, Mohamad Hamad, Ali El-Keblawy

**Affiliations:** 1 Department of Botany and Microbiology, Faculty of Science, Menoufia University, Shebeen El-Kom, Egypt; 2 Research Institute for Medical and Health Sciences, University of Sharjah, Sharjah, United Arab Emirates; 3 Department of Clinical Nutrition and Dietetics, College of Health Sciences, University of Sharjah, Sharjah, United Arab Emirates; 4 Department of Nutrition and Food Technology, Faculty of Agriculture, Jordan University of Science and Technology, Irbid, Jordan; 5 Department of Biotechnology, College of Sciences, Taif University, Taif, Saudi Arabia; 6 Department of Pharmaceutical Sciences, College of Pharmacy, Princess Nourah Bint Abdulrahman University, Riyadh, Saudi Arabia; 7 Department of Biology, College of Science, King Khalid University, Abha, Saudi Arabia; 8 Department of Medical Laboratory Sciences, College of Health Sciences, University of Sharjah, Sharjah, United Arab Emirates; 9 Department of Applied Biology, College of Sciences, University of Sharjah, Sharjah, United Arab Emirates; 10 Faculty of Pharmacy, Al Salam University, Tanta, Egypt

**Keywords:** bacteriophages, biocontrol, biofilm reduction, Escherichia coli O157:H7, food matrices, foodborne pathogens, *Salmonella enterica*

## Abstract

**Introduction:**

The application of bacteriophages in food systems requires evaluation under conditions that reflect realistic storage scenarios. This study evaluated two lytic bacteriophages, SW03 and CF03, targeting *Salmonella enterica* and Escherichia coli O157:H7, respectively, as potential biocontrol agents in food matrices.

**Methods:**

Phage SW03, isolated from municipal sewage, and phage CF03, isolated from camel feces, were assessed in artificially contaminated beef, chicken, and lettuce stored at 4 and 21 °C. High initial bacterial loads were used to simulate stringent contamination conditions. Antibacterial activity was evaluated during storage, and biofilm reduction was assessed using crystal violet staining. Key biological characteristics, including MOI, one-step growth, adsorption kinetics, and thermal stability, were also evaluated.

**Results:**

Both phages reduced bacterial populations across all tested matrices, with detectable antibacterial activity after 4 h and greater reductions after 24 h. The highest reductions were observed in lettuce samples at 21 °C, reaching approximately 4.0 and 3.5 log CFU g^−1^ for SW03 and CF03, respectively. At 4 °C, both phages retained measurable antibacterial activity, although reductions were lower and varied according to the food matrix. SW03 exhibited faster propagation and higher maximal titers than CF03, and both phages reduced preformed biofilm biomass.

**Discussion:**

These findings demonstrate the effectiveness of SW03 and CF03 under food-relevant storage conditions with high initial bacterial loads and support their potential use as biocontrol agents in food systems.

## 1 Introduction

Microbial contamination remains a major challenge in food safety and applied microbiology. Foods such as leafy vegetables, poultry, and red meat act as vehicles for the transmission of pathogenic bacteria during processing, storage, and distribution. Among the most important foodborne pathogens, *Salmonella enterica* and Shiga toxin–producing *Escherichia coli* (STEC), particularly E. coli O157:H7, are frequently implicated in outbreaks associated with contaminated animal-derived products and fresh produce (Scallan et al., 2011; World Health Organization (WHO), 2015; Franz and van Bruggen, 2008). These pathogens can survive under a wide range of environmental conditions and can persist on food surfaces or within processing environments, thereby increasing the risk of cross-contamination along the food production chain (Newell et al., 2010).

A major factor contributing to the persistence of foodborne pathogens is their ability to form biofilms. Biofilms are structured microbial communities embedded in extracellular polymeric substances that enhance resistance to environmental stress and antimicrobial treatments (Hall-Stoodley et al., 2004; Bridier et al., 2011). In food-processing and microbial environments, biofilm formation on surfaces and equipment can facilitate pathogen survival and cross-contamination, thereby complicating control strategies (Bridier et al., 2011; Giaouris et al., 2014). Conventional control methods, including thermal treatments, chemical sanitizers, and antibiotics, have limitations such as adverse effects on food quality, reduced efficacy in complex matrices, and the emergence of antimicrobial resistance (van Boekel et al., 2010; Hua et al., 2019; Ventola, 2015; Elbehiry et al., 2025). These challenges underscore the need for alternative strategies that are both effective and compatible with food safety and quality requirements.

In this context, bacteriophages (phages) have gained increasing attention as natural antimicrobial agents in microbiological and food safety applications for controlling foodborne pathogens. Numerous studies have demonstrated their effectiveness in reducing populations of *Salmonella*, *E. coli*, and *Listeria monocytogenes* in various food systems, typically achieving reductions of 1–3 log CFU g^−1^ depending on factors such as food matrix, phage characteristics, and storage conditions (Vikram et al., 2022; Shahdadi et al., 2024; Lasagabaster et al., 2020; Ferguson et al., 2013; Akdemir Evrendilek, 2026; Elbehiry and Alajaji, 2026; Pelyuntha and Vongkamjan, 2023; Jayamanne and Foddai, 2025). In addition, several phage-based products, including ListShield™, EcoShield™, and SalmoFresh™, have been granted Generally Recognized as Safe (GRAS) status by the FDA and are used as processing aids in food production in various countries, although this does not necessarily constitute regulatory approval in all jurisdictions (U.S. Food and Drug Administration, 2016; U.S. Food and Drug Administration, 2022; Food Safety and Inspection Service, 2024; Sulakvelidze, 2013; Bhardwaj et al., 2015). These findings highlight the potential of phages as practical tools for improving food safety. Phages offer several advantages, including high host specificity, self-replication at the site of infection, and minimal disruption to beneficial microbiota (Goodridge and Bisha, 2011; Sillankorva et al., 2012; Abedon et al., 2017). In addition to their activity against planktonic cells, phages have demonstrated the ability to disrupt bacterial biofilms through mechanisms such as cell lysis, enzymatic degradation of extracellular matrices, and repeated infection cycles (Chan and Abedon, 2015; Milho et al., 2019). However, phage efficacy in real food systems is highly variable and influenced by multiple environmental and application-related factors.

Key phage characteristics—including adsorption kinetics, latent period, burst size, multiplicity of infection (MOI), and environmental stability—play critical roles in determining antibacterial performance (Hyman and Abedon, 2009, 2010). Environmental factors such as temperature and physicochemical conditions can further influence both bacterial susceptibility and phage activity (Wdowiak et al., 2022). Despite extensive research on phage-based biocontrol, bacteriophage efficacy is known to vary depending on environmental conditions, application parameters, and experimental approaches, particularly in complex food systems (Ly-Chatain, 2014; Ribeiro et al., 2023).

Therefore, the present study aimed to evaluate the antibacterial efficacy of two strictly lytic bacteriophages, CF03 and SW03, targeting Escherichia coli O157:H7 and *Salmonella enterica*, respectively, under conditions relevant to food systems. Their antibacterial activity and biofilm biomass-reduction effects were assessed *in vitro* and in artificially contaminated food matrices, including plant- and animal-derived foods, under both room-temperature and refrigerated storage conditions. This study provides insights into the effectiveness of the two isolated bacteriophages under food-relevant conditions with elevated contamination levels and supports their application as biocontrol agents in food safety.

## 2 Materials and methods

### 2.1 Bacterial strains and culture conditions

Reference strains of Escherichia coli O157:H7 (strain 1934; Canadian Science Center for Human and Animal Health, Ottawa, Canada) and *Salmonella enterica* serovar Copenhagen PT 99 (Agriculture and Agri-Food Canada, Food Research Institute, Ottawa, Canada) were used as primary hosts for bacteriophage propagation and as model foodborne pathogens. Additional bacterial strains, including *Klebsiella pneumoniae* ATCC BAA-2146, *Acinetobacter baumannii* ATCC 19606, *Enterococcus faecalis* ATCC 29212, *Pseudomonas aeruginosa* ATCC 27853, and *Burkholderia cenocepacia* strain K56-2, were employed for host-range determination. All bacterial cultures were maintained on nutrient agar slants at 4 °C and routinely propagated in Luria–Bertani (LB) broth (Oxoid, United Kingdom) at 37 °C with shaking at 180 rpm prior to experimental use. Culture purity was verified microscopically and by plating before each assay as described by Hyman and Abedon (2009).

### 2.2 Bacteriophage preparation, amplification, and purification

Two strictly lytic bacteriophages, SW03 (GenBank accession no. PX705374.1) and CF03 (GenBank accession no. PX705375.1), were used in this study. Phage SW03 was isolated from municipal sewage and infects *Salmonella*, whereas CF03 was isolated from camel feces and infects *E. coli*. A concise summary of their genomic characteristics is provided to support their suitability for the application experiments.

All experiments were conducted using individual bacteriophages and their corresponding host strains. Phage SW03 was evaluated exclusively against *Salmonella*, while phage CF03 was tested against *E. coli*.

Phage propagation was performed using the double-layer agar method as described by Cao et al. (2024). Briefly, mid-log phase host bacterial cultures (OD₆₀₀ ≈ 0.3) were mixed with phage suspensions and molten soft agar (0.6% w/v) and then overlaid onto LB base agar plates (1.5% w/v; Oxoid, United Kingdom). Individual plaques were purified through three successive rounds of plating and subsequently eluted in SM buffer (50 mM Tris–HCl, pH 7.5; 100 mM NaCl; 10 mM MgSO₄; 0.01% gelatin; Sigma-Aldrich, United States). The resulting lysates were clarified by centrifugation (10,000 × g for 10 min at 4 °C), filtered through 0.22 μm membrane filters, and stored at 4 °C until use. Phage titers were determined using the double-layer agar (plaque assay) method and expressed as plaque-forming units per milliliter (PFU mL^−1^).

### 2.3 Antibacterial activity under food-related conditions

#### 2.3.1 Preparation of food samples

Fresh lettuce leaves, raw whole chicken, and beef slices (≈ 25 g each) were aseptically portioned and placed in sterile Petri dishes prior to inoculation (Ferguson et al., 2013; Duc et al., 2018).

#### 2.3.2 Inoculation of food samples and phage application

Overnight cultures of *E. coli* and *Salmonella* were adjusted to approximately 10^8^–10^9^ CFU mL^−1^. Food samples were surface-inoculated on both sides (0.1 mL per side; ≈ 10^8^–10^9^ CFU g^−1^) and left aseptically for 15 min at room temperature to facilitate bacterial attachment, without allowing complete surface drying. Phage suspensions with titers of approximately 10^7^–10^8^ PFU mL^−1^ were applied using the previously determined optimum MOI (0.1) for both SW03 and CF03. This value was selected based on prior MOI optimization experiments under laboratory conditions and used as the target phage-to-bacterium ratio for food application experiments. Each phage was applied to its corresponding host strain.

Untreated (bacteria-only), phage-only control (food samples treated with phage in the absence of bacteria), and sterile-water controls were included. The treatment volume did not exceed 2% of the total sample weight to minimize dilution effects (Ferguson et al., 2013; Kazi and Annapure, 2016). Samples were handled under aseptic conditions and analyzed immediately after treatment (time 0 h) and subsequently at 4 and 24 h during storage at 4 or 21 °C. Controls were processed in parallel to monitor bacterial growth in the absence of phage. Each treatment was performed using single phage–host pairs, and no mixed bacterial cultures were used.

Experiments were conducted under refrigerated (4 °C) and room temperature (21 °C) conditions, and samples were analyzed at designated time points (0, 4, and 24 h). An attachment period was included prior to phage application. The multiplicity of infection (MOI) was initially evaluated across multiple values, and an MOI of 0.1 was selected for subsequent experiments.

#### 2.3.3 Quantification of bacterial populations

Each 25 g food sample was homogenized with 225 mL of buffered peptone water (Oxoid, United Kingdom) using a stomacher for 2 min. The same homogenization and recovery procedure was applied consistently across all treated and untreated samples to ensure comparability between experimental conditions. Serial decimal dilutions were prepared, and appropriate dilutions were plated onto selective media. Xylose Lysine Deoxycholate (XLD) agar (Oxoid, United Kingdom) was used for the enumeration of *Salmonella*, while MacConkey agar (Oxoid, United Kingdom) was used for *E. coli*. Plates were incubated at 37 °C for 24 h, and colonies were subsequently counted. Results were expressed as colony-forming units per gram (CFU g^−1^) of sample (Pelyuntha and Vongkamjan, 2023).

#### 2.3.4 Evaluation of bacterial reduction and statistical analysis

Bacterial reduction was expressed as log₁₀ reduction calculated using the equation log₁₀(N₀/Nₜ), where N₀ represents the bacterial count in untreated control samples and N_t_ represents the bacterial count in the corresponding phage-treated samples. All experiments were conducted in triplicate using independently prepared food samples, and results are presented as mean ± standard deviation (SD). Statistical analysis was performed using two-way analysis of variance (ANOVA) to evaluate the effects of food matrix and storage condition on bacterial reduction. Analyses were conducted using GraphPad Prism version 10 (GraphPad Software, San Diego, CA, United States), and statistical significance was considered at *p* < 0.05.

### 2.4 Biofilm reduction assay (*in vitro*)

#### 2.4.1 Biofilm formation

Biofilm formation was evaluated using a crystal violet microtiter plate assay with minor modifications, as previously described by Yin et al. (2019) and Taj et al. (2024), using crystal violet (Sigma-Aldrich, United States). The biofilm-forming ability of *E. coli* and *Salmonella* was assessed.

Overnight bacterial cultures were adjusted to approximately 1 × 10^6^ CFU mL^−1^, and 200 μL aliquots were dispensed into sterile 96-well flat-bottom microtiter plates. Peripheral wells were filled with sterile distilled water to minimize evaporation, while LB medium served as a negative control. Plates were incubated statically at 37 °C for 24 h to allow biofilm development. The 24 h incubation period was selected based on previous studies demonstrating that this duration is sufficient for the formation of stable and mature biofilms under static conditions (Gungor et al., 2024).

Following incubation, planktonic cells were removed, and wells were gently washed twice with sterile phosphate-buffered saline (PBS; Sigma-Aldrich, United States). The remaining biofilms were fixed with 200 μL of 95% ethanol (Merck, Germany) for 15 min and air-dried. Subsequently, 200 μL of 0.1% (w/v) crystal violet solution was added to each well and incubated for 15 min at room temperature. Excess stain was removed by rinsing the wells with distilled water, and the plates were allowed to dry. The bound dye was then solubilized using 200 μL of 95% ethanol, and biofilm biomass was quantified by measuring absorbance at 595 nm using a microplate reader.

#### 2.4.2 Phage treatment of preformed biofilms

For evaluation of biofilm reduction, preformed biofilms were established by incubating bacterial cultures in 96-well microtiter plates at 37 °C for 24 h under static conditions. After incubation, culture supernatants were removed, and wells were gently washed three times with sterile PBS to remove planktonic cells.

Subsequently, 200 μL of purified phage suspensions (10^8^ PFU mL^−1^) of SW03 or CF03 were added to the respective wells, while fresh LB medium served as the untreated control (preformed biofilm without phage treatment) (Duc et al., 2018). Plates were incubated at 37 °C for 24 h. Following incubation, supernatants were removed, and wells were gently washed twice with sterile PBS. The remaining biofilms were fixed with 95% ethanol for 15 min, stained with 0.1% (w/v) crystal violet, and quantified by measuring absorbance at 595 nm using a microplate reader.

#### 2.4.3 Quantification of biofilm reduction

Biofilm reduction (%) was calculated using the following equation, based on untreated preformed biofilms as the reference condition:

Biofilm reduction (%) = (OD_control − OD_treated)/OD_control × 100.

All experiments were performed in triplicate, and blank wells were included for background correction prior to absorbance measurements. It should be noted that crystal violet staining quantifies total biofilm biomass and does not provide a direct measure of viable cells.

### 2.5 Biological characterization of bacteriophages

To comprehensively evaluate the biological characteristics of the bacteriophages, a series of standard assays was performed, including MOI optimization, one-step growth analysis, adsorption kinetics, and thermal stability assessment.

#### 2.5.1 Effect of multiplicity of infection on phage production

To evaluate the effect of infection density on phage production, phage yields were measured at different multiplicities of infection (MOI: 10, 1, 0.1, 0.01, and 0.001). The MOI that produced the highest phage titer was selected as the optimal MOI for subsequent experiments, consistent with previously described approaches for MOI optimization (Hyman and Abedon, 2009). MOI values were calculated separately for each phage–host pair based on the corresponding bacterial concentration and phage titer.

#### 2.5.2 One-step growth analysis

Replication kinetics of the bacteriophages were determined using a standard one-step growth assay following the method described by Hyman and Abedon (2009). Mid-log phase host bacterial cultures were infected with the corresponding bacteriophage (SW03 or CF03) at an MOI of 0.01 for one-step growth analysis, following standard phage-growth assay conditions. The phage–host mixtures were allowed to adsorb for 10 min at 37 °C. Subsequently, the infected cultures were centrifuged and washed to remove unadsorbed phages, then resuspended and incubated at 37 °C for 120 min. Samples were collected at 10 min intervals, and phage titers were determined using the plaque assay. The latent period and burst size were estimated from the resulting growth curves according to the classical Ellis–Delbrück model (Ellis and Delbrück, 1939).

#### 2.5.3 Adsorption kinetics

Phage–host mixtures were prepared using predetermined multiplicities of infection, with SW03 applied at MOI values ranging from 0.1 to 1, and CF03 at an MOI of 0.1. Phage titers and bacterial concentrations were standardized prior to infection to ensure consistent initial conditions. Samples (1 mL) were collected at 2 min intervals for 20 min, immediately centrifuged (6,000 × g, 2 min, 4 °C), and the supernatants were assayed for unadsorbed phages using the double-layer agar plaque assay. The percentage of adsorption was calculated based on the reduction in free phage titers relative to the initial titer at time zero. The adsorption rate constant (k) was determined according to the method described by Hyman and Abedon (2010).

#### 2.5.4 Thermal stability assessment

Thermal tolerance of the phages was assessed by incubating phage suspensions (approximately 10^9^ PFU mL^−1^) at 40, 50, 60, 70, 80, 90, and 100 °C for 30 min in sealed tubes using a calibrated water bath. After incubation, samples were rapidly cooled on ice to halt thermal inactivation. The number of surviving phage particles was quantified by plaque assay, and thermal stability was expressed as log₁₀(PFU_t_/PFU₀) according to standard thermal inactivation models (Kim et al., 2024).

## 3 Results

The antibacterial performance of bacteriophages SW03 and CF03 was evaluated across different food matrices and experimental conditions. Both phages significantly suppressed bacterial growth and reduced preformed biofilm biomass, although their efficacy varied depending on the food matrix and environmental conditions.

### 3.1 Genomic and morphological characterization of phages SW03 and CF03

To provide essential background for the application-focused experiments, a concise summary of the genomic characteristics of phages SW03 and CF03 is presented. Transmission electron microscopy (TEM) was used to examine phage morphology, while plaque morphology analysis showed clear, well-defined plaques without halo formation. Together with genomic analysis confirming the absence of lysogeny-associated genes, these findings support the lytic nature of phages SW03 and CF03.

The genome of SW03 is 85,005 bp, with a GC content of 43.8%, and encodes 141 predicted coding sequences (CDSs), including 23 tRNA genes. In contrast, CF03 has a genome size of 77,673 bp, a GC content of 40.9%, and encodes 92 predicted features and 3 tRNA genes.

Genome annotation was performed using the RAST server. No genes associated with lysogeny, such as integrases, or with virulence factors or antibiotic-resistance determinants were detected, further supporting their strictly lytic lifestyle and their suitability for food applications in terms of biosafety. Functional annotation indicated that the majority of predicted genes are associated with structural components, DNA replication, and host lysis.

The genome sequences of SW03 and CF03 have been deposited in GenBank under accession numbers PX705374.1 and PX705375.1, respectively. Based on genomic classification, both phages belong to the class Caudoviricetes. These characteristics further support the suitability of both phages for subsequent biological evaluation and application in food systems.

Host-range screening further showed that the tested phages exhibited infectivity within the Enterobacteriaceae group. SW03 showed activity against *Salmonella enterica* strains and also infected *Klebsiella pneumoniae*, whereas CF03 showed activity against Escherichia coli O157:H7 strains. No lytic activity was observed against the tested unrelated bacterial species, including *Acinetobacter baumannii*, *Enterococcus faecalis, Pseudomonas aeruginosa*, and *Burkholderia cenocepacia*.

### 3.2 Evaluation of phage efficacy in food systems

#### 3.2.1 Temporal dynamics of bacterial reduction in food samples

**Figure 1:**
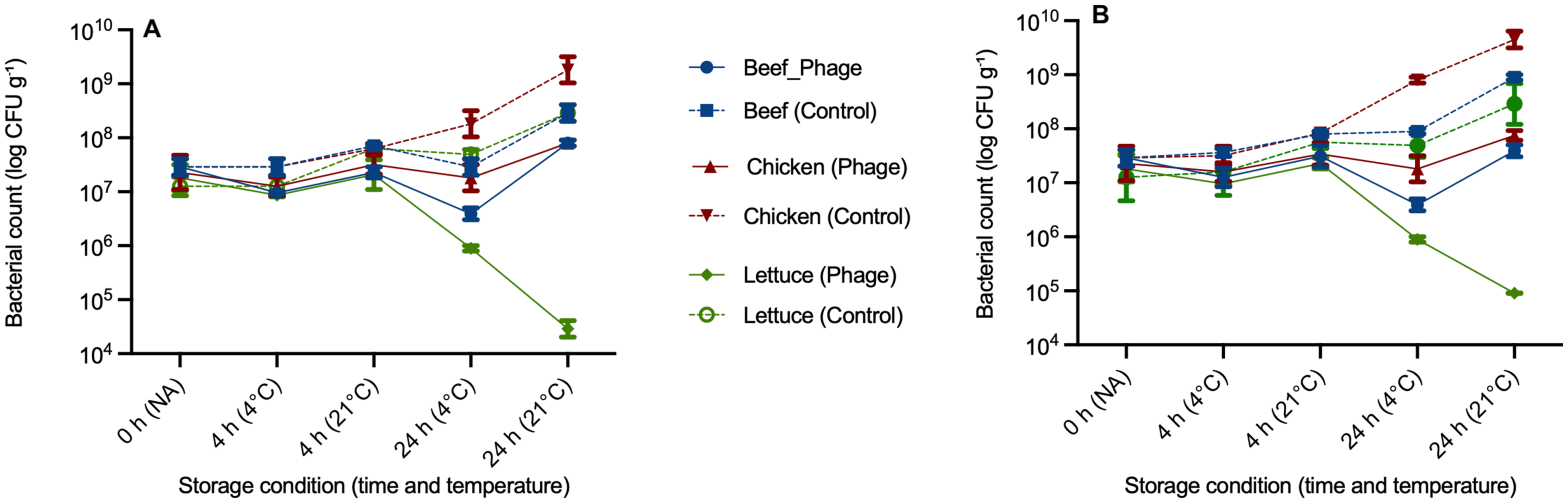
Changes in bacterial counts in food matrices following bacteriophage treatment under different storage conditions. **(A)** Antibacterial activity of phage SW03 in beef, chicken, and lettuce samples stored at 0, 4, and 24 h. **(B)** Antibacterial activity of phage CF03 under the same conditions. Each phage was tested against its corresponding host strain. Data are presented as mean bacterial counts (log CFU g^−1^) ± SD (*n* = 3), and error bars represent standard deviation. The *y*-axis is presented on a logarithmic scale.

At the whole-food level, the antibacterial efficacy of bacteriophages SW03 and CF03 was evaluated by monitoring temporal changes in bacterial populations under different storage conditions (Figure 1).

Baseline bacterial counts in food samples prior to inoculation were low or below the detection limit (≤10^2^ CFU g^−1^). Following inoculation, bacterial levels increased to approximately 10^8^–10^9^ CFU g^−1^ at time zero, confirming successful establishment of the target bacterial populations. Intermediate evaluation at 4 h demonstrated detectable antibacterial activity in all tested food matrices under both refrigerated (4 °C) and room temperature (21 °C) conditions, although reductions were generally lower than those observed after 24 h. After 24 h, untreated samples showed sustained or increased bacterial counts, whereas phage-treated samples exhibited significant reductions relative to the corresponding controls.

Under refrigerated conditions (4 °C), both phages reduced bacterial counts across all food matrices, with generally moderate reductions. Detectable antibacterial activity was also observed at 4 h, with reductions ranging from approximately 0.17–0.48 log CFU g^−1^ depending on the food matrix and phage–host system. CF03 showed higher reductions than SW03 in beef (≈1.36 vs. 0.87 log CFU g^−1^) and chicken (≈1.64 vs. 1.00 log CFU g^−1^), while both phages exhibited comparable activity in lettuce (≈1.74 log CFU g^−1^).

At room temperature (21 °C), higher variability in antibacterial efficacy was observed. At 4 h, measurable reductions were also detected under these conditions, ranging from approximately 0.29–0.49 log CFU g^−1^ across the tested food matrices. In beef, CF03 again showed higher reductions (≈1.36 log CFU g^−1^) compared with SW03 (≈0.56 log CFU g^−1^). In contrast, SW03 showed greater reductions in chicken (≈1.36 log CFU g^−1^) than CF03 (≈0.77 log CFU g^−1^). The highest reductions were observed in lettuce, where SW03 reached approximately 4 log CFU g^−1^ reduction, whereas CF03 reached approximately 3.5 log CFU g^−1^ after 24 h.

Two-way ANOVA indicated that bacterial reduction was significantly influenced by both the food matrix and storage conditions, with a significant interaction between these factors (*p* < 0.0001).

#### 3.2.2 Comparative antibacterial efficacy across food matrices

**Figure 2:**
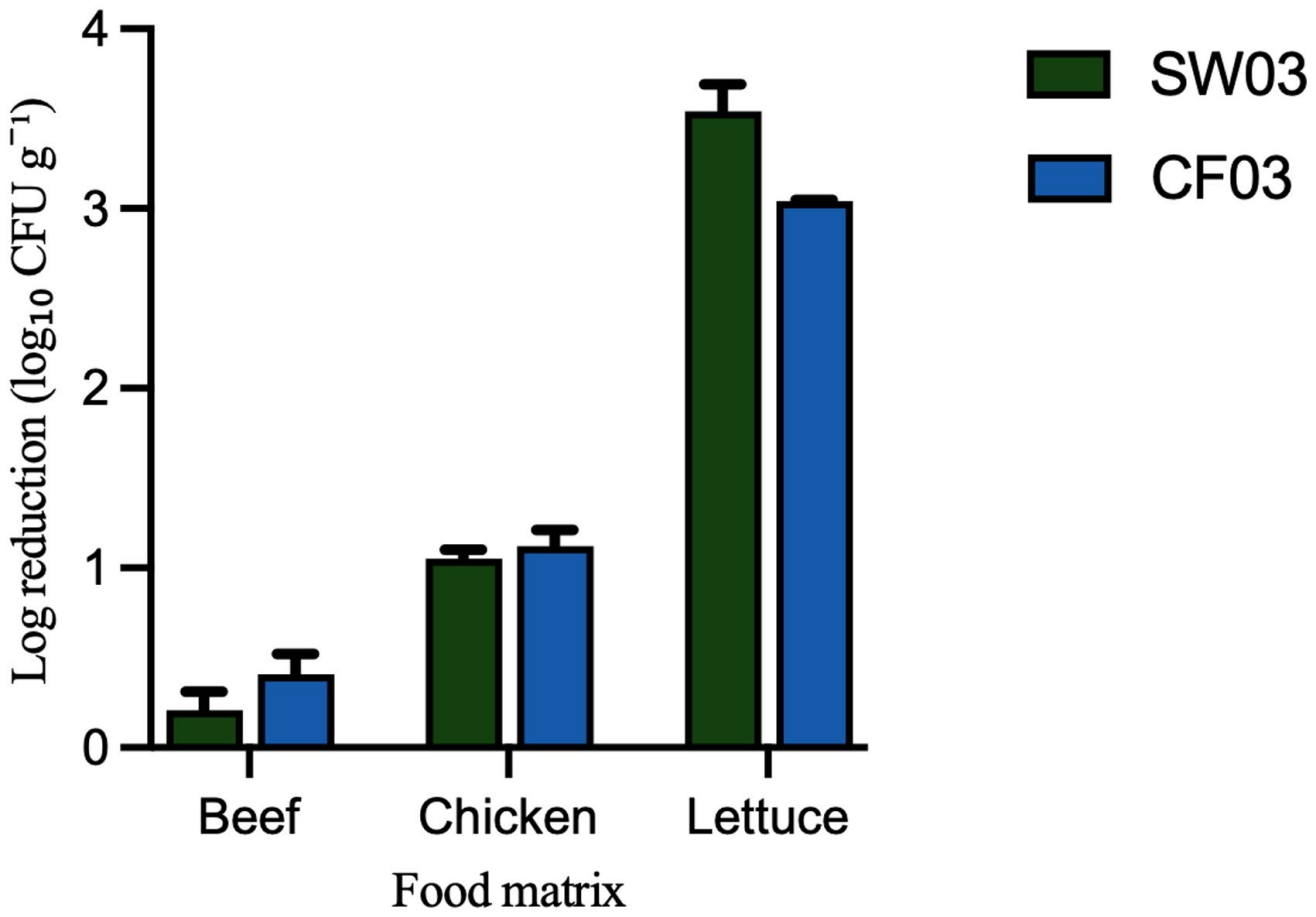
Mean endpoint bacterial reductions achieved by bacteriophages SW03 and CF03 across different food matrices. Log reductions (log_10_ CFU g^−1^) were calculated relative to the initial inoculum levels (10^8^ CFU g^−1^ for beef and lettuce, and 10^9^ CFU g^−1^ for chicken) after 24 h under the tested conditions. Bars represent mean values ± SD (*n* = 3) from three independent experiments, and error bars indicate standard deviation.

To enable comparative assessment of bacteriophage efficacy across different food matrices, antibacterial effects were quantified as log reductions after 24 h at 21 °C (Figure 2), the condition associated with the highest observed activity.

Mean endpoint reductions revealed matrix-dependent differences in bacteriophage efficacy across the tested food samples relative to the initial inoculum levels.

In beef samples (initial inoculum 10^8^ CFU g^−1^), reductions were lower, with SW03 showing an average reduction of approximately 0.21 ± 0.10 log CFU g^−1^ and CF03 showing approximately 0.41 ± 0.11 log CFU g^−1^.

In chicken samples (initial inoculum 10^9^ CFU g^−1^), both phages showed comparable reductions, with mean decreases of approximately 1.05 ± 0.05 log CFU g^−1^ for SW03 and 1.12 ± 0.09 log CFU g^−1^ for CF03.

In lettuce samples (initial inoculum 10^8^ CFU g^−1^), phage SW03 showed an average reduction of approximately 3.54 ± 0.15 log CFU g^−1^, while CF03 showed a reduction of approximately 3.04 ± 0.01 log CFU g^−1^.

These findings complement the temporal trends observed earlier, further highlighting the influence of food matrix characteristics on phage performance.

Two-way ANOVA indicated that bacterial reduction was significantly influenced by the food matrix (*p* < 0.0001), while the effect of phage type was not statistically significant (*p* = 0.1278), although a significant interaction between factors was also observed (*p* < 0.0001).

#### 3.2.3 Effect of storage conditions on phage efficacy across food matrices

**Figure 3:**
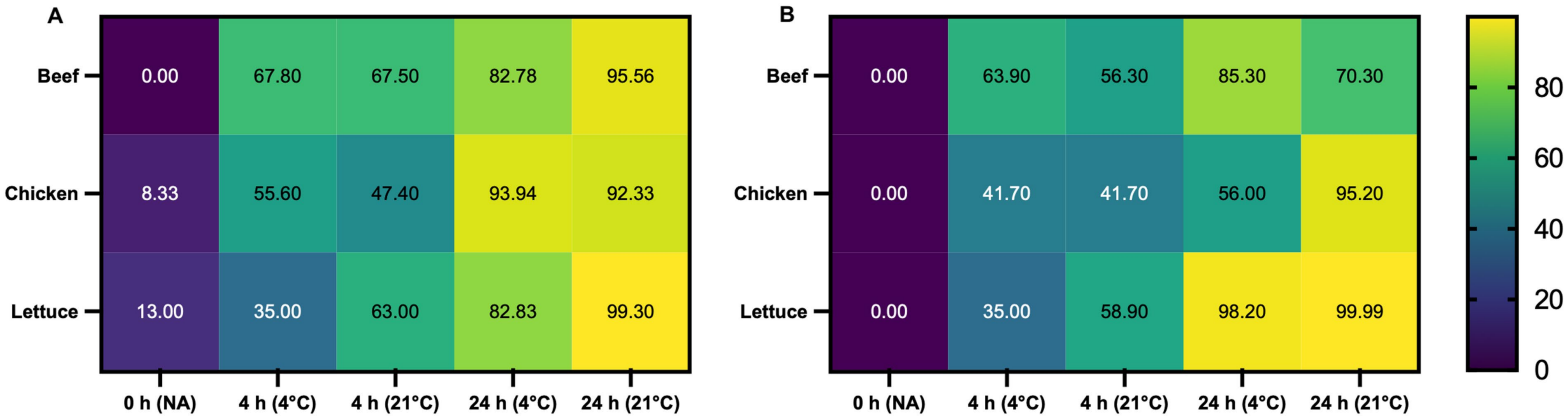
Effect of storage conditions and food matrix on bacterial reduction following bacteriophage treatment. Heat maps illustrate the percentage reduction of bacterial populations in beef, chicken, and lettuce samples treated with bacteriophages SW03 **(A)** and CF03 **(B)**. Reductions were assessed at 0, 4, and 24 h of storage at 4 and 21 °C. Color intensity represents the magnitude of bacterial reduction (%). Values represent mean percentages (*n* = 3) from independent experiments.

Temperature and matrix composition affected bacterial reduction across the tested food matrices (Figure 3). To further visualize reduction patterns across food matrices and temperature conditions, the data are presented as percentage reductions in a heat map format.

Detectable reductions were also observed at 4 h under both refrigerated and room temperature conditions for both phages, although reduction levels were generally lower than those detected after 24 h. For SW03 (Figure 3A), reductions ranged from 35.00 to 67.80% at 4 °C and from 47.40 to 67.50% at 21 °C. Similarly, CF03 (Figure 3B) showed reductions ranging from 35.00 to 63.90% under refrigerated conditions and from 41.70 to 58.90% at room temperature after 4 h.

Under refrigerated storage (4 °C), reduction levels varied across food matrices. For SW03 (Figure 3A), reductions reached 82.78% in beef, 93.94% in chicken, and 82.83% in lettuce. Likewise, CF03 (Figure 3B) achieved reductions of 85.30% in beef, 56.00% in chicken, and 98.20% in lettuce.

After 24 h at 21 °C, both phages generally produced higher levels of bacterial reduction. As shown in Figure 3A, phage SW03 achieved reductions of 95.56% in beef, 92.33% in chicken, and 99.30% in lettuce. Similarly, Figure 3B shows that phage CF03 produced reductions of 70.30% in beef, 95.20% in chicken, and 99.99% in lettuce.

Higher reductions were generally observed at 21 °C compared with 4 °C, although some matrix-dependent variations were noted, particularly in chicken samples for SW03 and beef samples for CF03.

### 3.3 Antibiofilm activity against preformed biofilms

As shown in Table 1, bacteriophage treatment resulted in measurable reductions in preformed biofilms. Phage SW03 showed a decrease in OD595 values from 0.344 ± 0.040 to 0.073 ± 0.005. Similarly, phage CF03 showed a reduction from 0.306 ± 0.021 to 0.106 ± 0.012 following treatment.

**Table 1:** Effect of bacteriophage treatment on preformed biofilms determined by crystal violet assay (OD595).

Phage	OD595 before treatment (mean ± SD)	OD595 after treatment (mean ± SD)
SW03	0.344 ± 0.040	0.073 ± 0.005
CF03	0.306 ± 0.021	0.106 ± 0.012

Preformed biofilms were developed in 96-well microtiter plates and treated with bacteriophages SW03 or CF03. Biofilm biomass was quantified before and after phage treatment using the crystal violet assay, and absorbance was measured at 595 nm. Values represent mean ± standard deviation (SD) of three independent replicates (*n* = 3). Untreated preformed biofilms were used as the reference condition.

**Figure 4:**
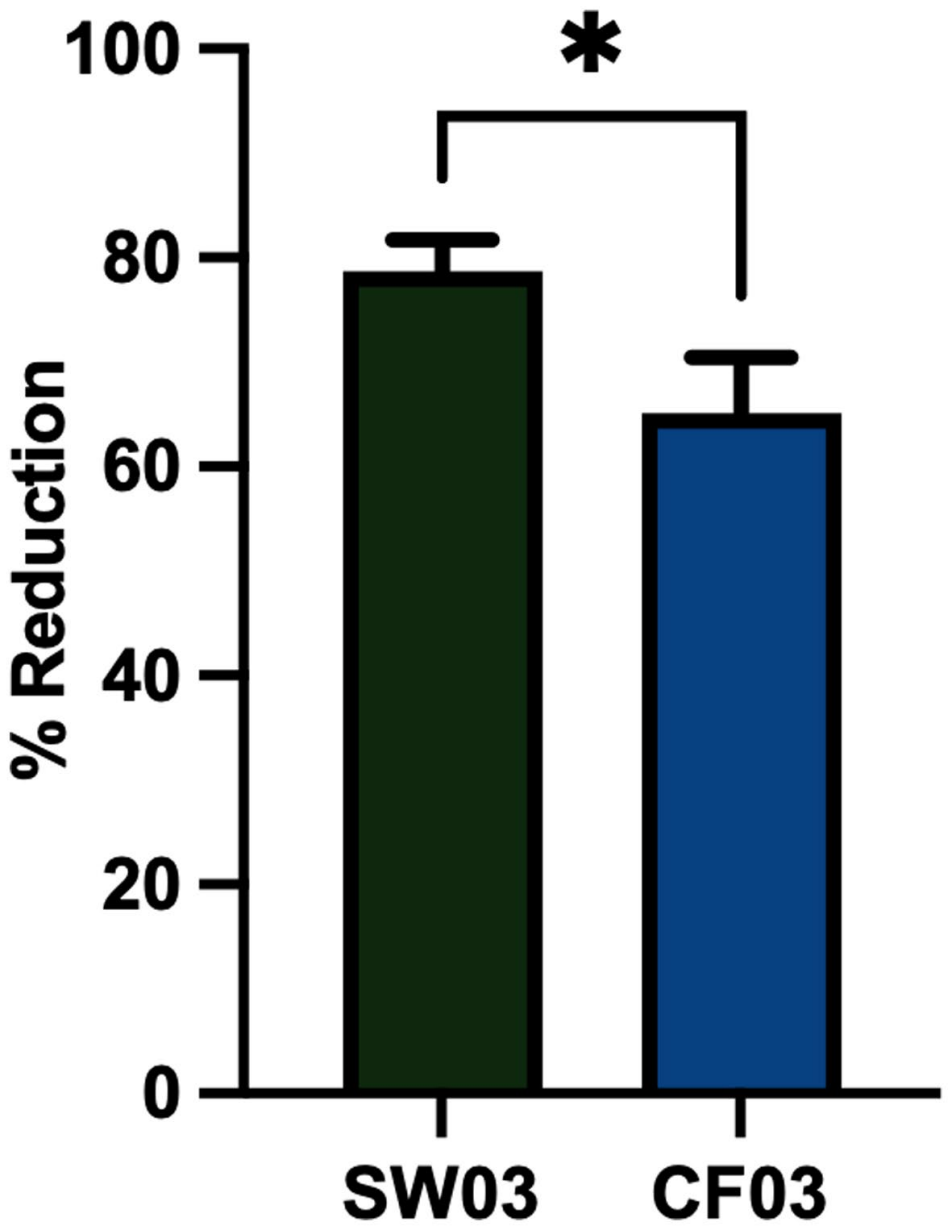
Antibiofilm activity of bacteriophages SW03 and CF03 against preformed bacterial biofilms. Biofilm biomass was quantified using the crystal violet microtiter plate assay, and absorbance was measured at 595 nm before and after phage treatment. Bars represent the mean percentage reduction in biofilm biomass ± SD (*n* = 3). *indicates a statistically significant difference between SW03 and CF03 (*p* < 0.05).

Calculated biofilm biomass reduction percentages were approximately 78.7% for SW03 and 65.2% for CF03. These results are further illustrated in Figure 4. The difference between the two phages was statistically significant (*p* = 0.0283), with SW03 producing a greater reduction in crystal-violet-stained biofilm biomass than CF03.

### 3.4 Biological characteristics supporting phage activity

To provide a biological context for the observed antibacterial activity, key properties of bacteriophages SW03 and CF03 were evaluated.

#### 3.4.1 Effect of multiplicity of infection on phage productivity

**Figure 5:**
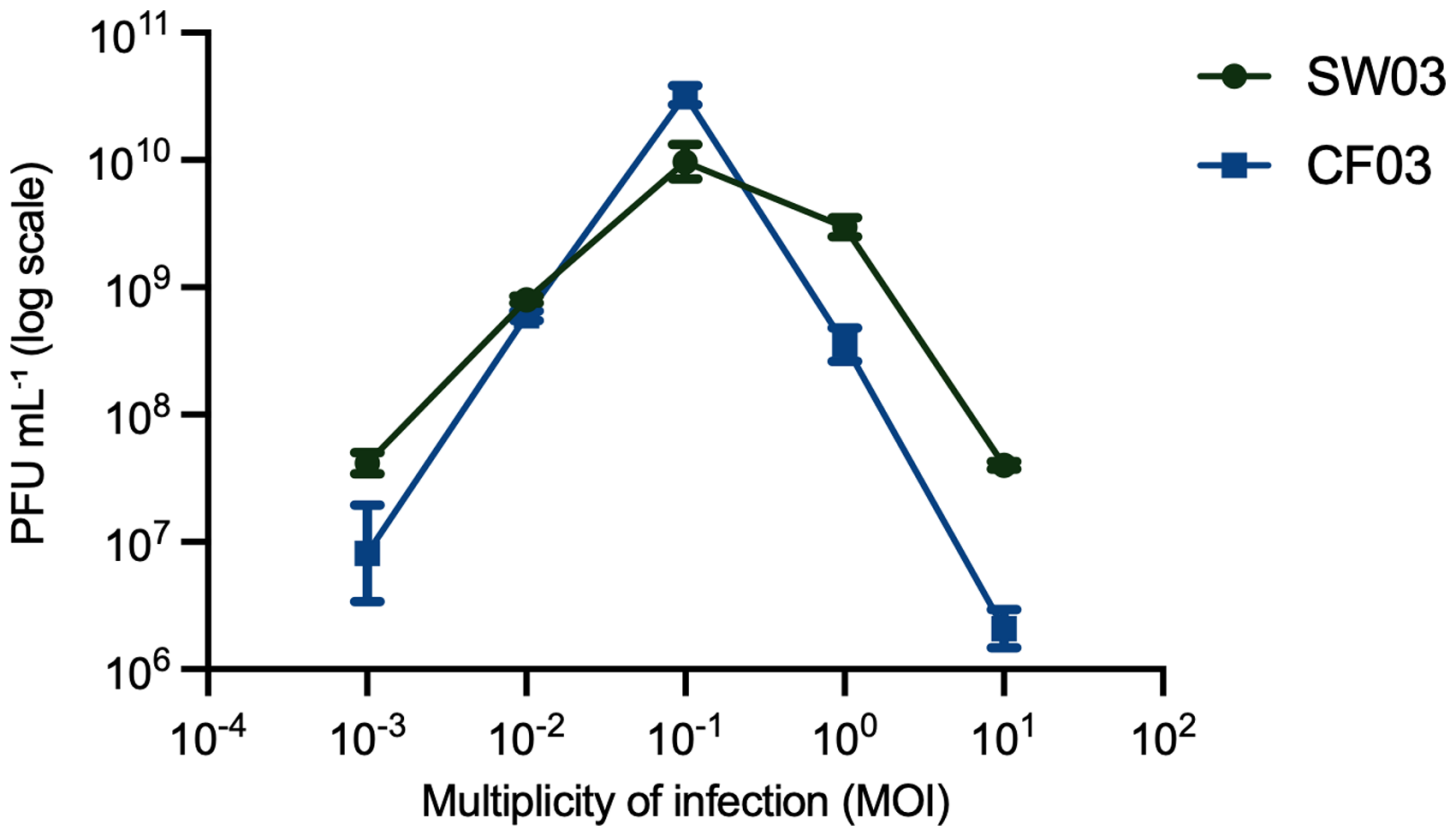
Effect of MOI on phage production. Phage titers (PFU mL^−1^) of SW03 and CF03 were measured across MOI values ranging from 0.001 to 10. Data are presented as mean phage titers ± SD (*n* = 3), and error bars represent standard deviation.

MOI assays showed a bell-shaped pattern of phage production, with maximum yields observed at intermediate MOI values (0.1–1) (Figure 5), which were considered optimal under the tested conditions. For phage SW03, the highest titers were obtained at MOI 0.1–1, reaching approximately 7.0 × 10^9^ to 1.3 × 10^1^⁰ PFU mL^−1^. In contrast, phage CF03 showed its highest production at MOI 0.1, with titers ranging from approximately 2.7 × 10^1^⁰ to 3.8 × 10^1^⁰ PFU mL^−1^. At lower MOIs (0.001) and higher MOIs (10), phage yields were reduced for both phages, typically by approximately one order of magnitude compared with peak production levels.

#### 3.4.2 One-step growth characteristics

**Figure 6:**
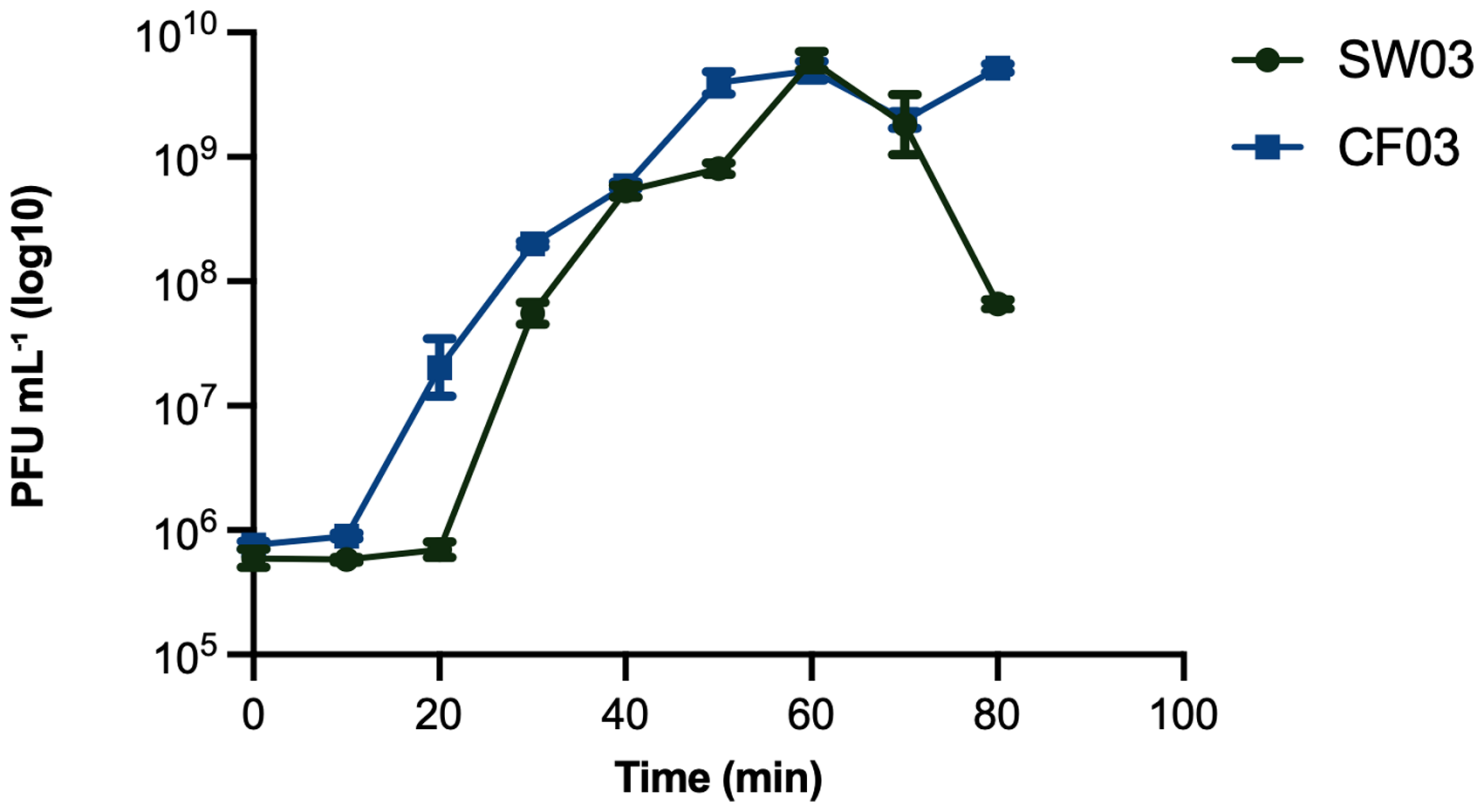
One-step growth curves of bacteriophages SW03 and CF03. Phage titers (PFU mL^−1^) were measured over time following infection of host bacterial cultures. Data are presented as mean values ± SD (*n* = 3), and error bars represent standard deviation.

As shown in Figure 6, both phages exhibited a typical one-step growth pattern, characterized by an initial latent phase followed by an increase in phage titers. Phage SW03 showed a latent period of approximately 20–30 min, after which phage titers increased, reaching peak values of approximately 6–7 × 10^9^ PFU mL^−1^ at 60 min. In contrast, phage CF03 reached peak titers between 50 and 60 min, with values of approximately 4–6 × 10^9^ PFU mL^−1^. The estimated burst size was approximately 10^2^ PFU per infected cell for both SW03 and CF03.

Following the peak phase, phage titers generally declined over time, with minor fluctuations observed, particularly for CF03. These data provide a biological context for interpreting the observed antibacterial performance of the phages under food-related conditions.

#### 3.4.3 Adsorption behavior of phages

**Figure 7:**
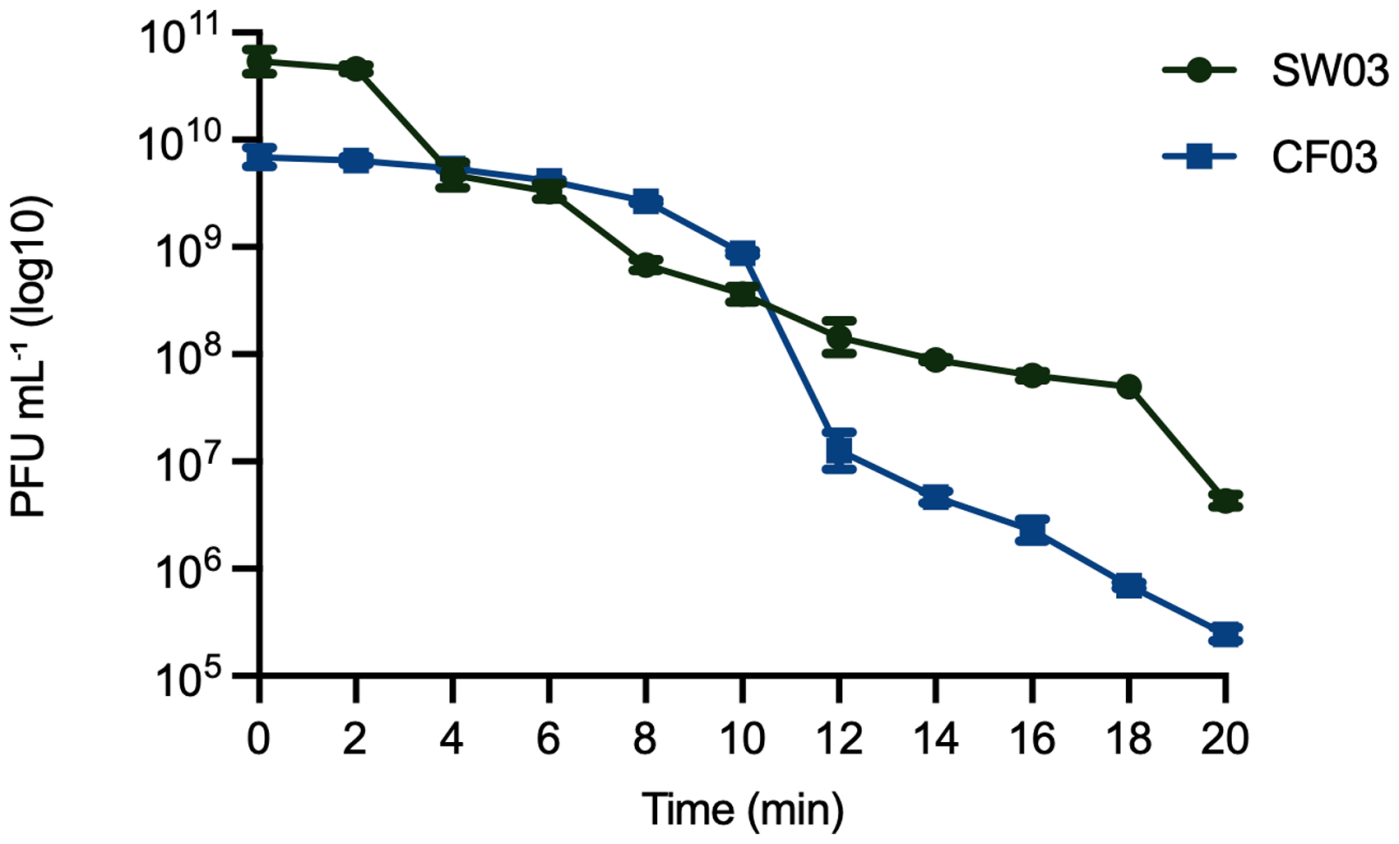
Adsorption kinetics of bacteriophages SW03 and CF03. The number of free phage particles (PFU mL^−1^) was measured over time following infection of host bacterial cells. Data are presented as mean values ± SD (*n* = 3), and error bars represent standard deviation.

Adsorption kinetics analysis (Figure 7) showed a decline in free phage particles over time following infection. Phage SW03 exhibited faster adsorption dynamics, with approximately 16% of particles adsorbing within the first 10 min and nearly 90% adsorption achieved within approximately 20 min. In contrast, phage CF03 showed slower initial adsorption, with only about 9% adsorption after 10 min and approximately 23% after 20 min. These values were calculated based on the reduction in free phage titers relative to the initial titer at time zero. Over the full adsorption period, both phages showed a reduction in free phage titers, corresponding to adsorption to host cells. Differences in adsorption rates between the two phages were observed under the tested conditions.

#### 3.4.4 Thermal stability

**Figure 8:**
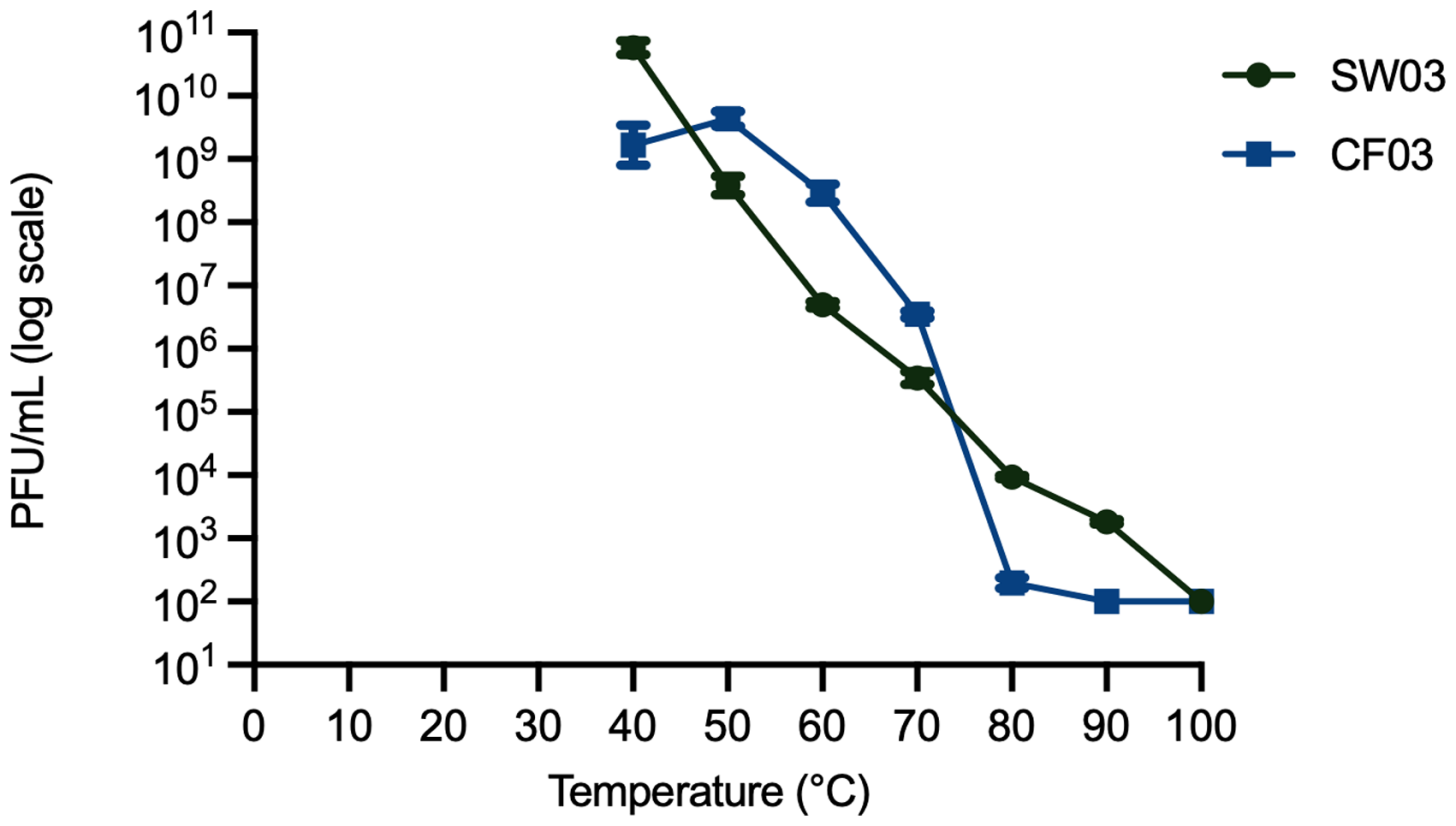
Thermal stability of bacteriophages SW03 and CF03. Phage suspensions were exposed to increasing temperatures (40–100 °C), and the remaining infective particles (PFU mL^−1^) were quantified to evaluate thermal tolerance. Both phages remained relatively stable at 40 °C but exhibited progressively reduced infectivity with increasing temperature. Data are presented as mean phage titers ± SD (*n* = 3), and error bars represent standard deviation.

Thermal stability analysis showed a progressive decline in phage infectivity with increasing temperature (Figure 8). At 40 °C, both phages maintained high titers, indicating substantial stability under mild heating conditions. As temperature increased to 50 °C, phage SW03 titers decreased markedly, declining from approximately 5–7 × 10^1^⁰ PFU mL^−1^ to about 3–5 × 10^8^ PFU mL^−1^, while CF03 showed a slight increase in titer (approximately 3–5 × 10^9^ PFU mL^−1^) compared with 40 °C, representing a minor variation at this intermediate temperature, followed by a sharp decline at higher temperatures. Further temperature increases resulted in rapid loss of infectivity for both phages. At 60 °C, phage titers dropped to approximately 10^6^ PFU mL^−1^ for SW03 and about 10^8^ PFU mL^−1^ for CF03. At 70 °C, only residual activity remained, with titers declining to approximately 10^5^ PFU mL^−1^ for SW03 and approximately 10^6^ PFU mL^−1^ for CF03. Exposure to 80 °C and higher temperatures caused near-complete inactivation, with only trace levels of detectable phage particles remaining. Both phages exhibited reduced infectivity with increasing temperature, with only minor variations observed at intermediate temperatures.

## 4 Discussion

### 4.1 Antibacterial activity in food systems

This study shows that the lytic bacteriophages SW03 and CF03 reduced their corresponding bacterial targets across different food matrices under storage conditions, although the magnitude of reduction depended strongly on the matrix and temperature. Both phages demonstrated measurable bacterial reduction in beef, chicken, and lettuce samples, supporting previous reports on the potential of bacteriophages as targeted biocontrol agents in food environments (Sillankorva et al., 2012; Abedon et al., 2017; Pelyuntha and Vongkamjan, 2023).

To ensure the safety of these application-oriented experiments, both phages were characterized at the genomic level. Genomic analysis confirmed the absence of genes associated with lysogeny, virulence factors, and antibiotic resistance, which are critical criteria for bacteriophage use in food systems (EFSA Panel on Food Contact Materials, Enzymes, Flavourings and Processing Aids (CEF), 2016; Garcia et al., 2008). Functional annotation further indicated that the identified genes are primarily involved in structural assembly, DNA replication, and host lysis, consistent with a strictly lytic lifestyle.

Phage application in artificially contaminated food samples confirmed effective bacterial reduction across beef, chicken, and lettuce, although the magnitude of reduction varied depending on the food matrix. Similar matrix-dependent variations in phage efficacy have been reported previously in food application studies. Variations in food surface characteristics, moisture availability, and matrix complexity may influence phage distribution and accessibility to bacterial targets. In particular, solid and structurally complex food matrices such as meat products may physically limit phage diffusion and restrict phage–host contact efficiency, whereas more exposed surfaces such as leafy vegetables may facilitate greater accessibility of phage particles to bacterial cells. These factors may contribute to the higher reductions observed in lettuce samples compared with beef and chicken in the present study (Guenther et al., 2009; Pelyuntha and Vongkamjan, 2023).

The phages demonstrated infectivity against multiple strains within the Enterobacteriaceae group during host range screening, including several Escherichia coli O157:H7 and *Salmonella enterica* strains. In addition, SW03 exhibited infectivity against *Klebsiella pneumoniae*. No activity was observed against unrelated bacterial species, including *Acinetobacter baumannii*, *Enterococcus faecalis*, *Pseudomonas aeruginosa*, and *Burkholderia cenocepacia*. Although food application experiments were conducted using single representative host strains, the observed infectivity patterns support the potential applicability of both phages in food systems (Garcia et al., 2008).

### 4.2 Antibiofilm activity

The crystal violet biofilm assays demonstrated measurable reductions in preformed biofilm biomass following bacteriophage treatment, with SW03 showing stronger activity than CF03 and resulting in greater biomass reduction than CF03. Biofilms are known to enhance bacterial resistance to antimicrobial treatments, making them a significant concern in food-processing environments (Giaouris et al., 2014). Similar reductions in biofilm biomass following bacteriophage treatment have been reported previously against biofilm-associated bacteria, including *Escherichia coli*, *Klebsiella pneumoniae*, and other foodborne pathogens (Pallavali et al., 2021; El-Atrees et al., 2022; Gutiérrez et al., 2018). The antibiofilm activity observed in the present study is therefore consistent with previous reports demonstrating the potential of lytic bacteriophages to reduce biofilm-associated bacterial populations. The greater biomass reduction observed with SW03 may indicate more effective phage–host interactions under the tested conditions. These findings further support the potential use of lytic bacteriophages as complementary tools to reduce biofilm-associated bacterial contamination in food-related environments.

### 4.3 Biological interpretation of phage characteristics

Biological characterization provided insight into the dynamics of phage–host interactions. The observation that maximal phage production occurred at intermediate MOI values suggests that balanced phage–host ratios are important for efficient phage propagation under the tested conditions. Similar optimal MOI ranges have been reported previously for lytic bacteriophages targeting *Escherichia coli*, in which maximal phage proliferation often occurs at intermediate infection densities (Sada and Tessema, 2024). This behavior is consistent with classical infection-density principles, in which low MOI values may limit productive infection because of insufficient phage–host encounters, while excessively high MOIs may reduce overall propagation efficiency due to rapid host lysis or limited availability of uninfected host cells (Hyman and Abedon, 2009).

One-step growth analysis confirmed a typical lytic replication cycle for both phages, with latent periods occurring within a comparable time range. However, SW03 reached slightly higher peak titers than CF03, suggesting more efficient propagation under the tested conditions. Similar one-step growth characteristics, including latent periods ranging between 20 and 30 min followed by rapid increases in phage titers, have been reported previously for lytic bacteriophages targeting pathogenic bacteria (Rezaei et al., 2022). In addition, the estimated burst sizes observed for SW03 and CF03 (~10^2^ PFU per infected cell) are comparable to values previously reported for lytic bacteriophages targeting *Escherichia coli*, where burst sizes ranging from approximately 10^2^ to 2 × 10^2^ PFU per infected cell were observed (Sada and Tessema, 2024). Such replication characteristics are generally considered advantageous for rapid bacterial control and efficient phage propagation (Hyman and Abedon, 2009).

Adsorption assays indicated an efficient interaction between phages and their host cells under the tested conditions, which is an important characteristic for successful bacteriophage application in food systems. Efficient adsorption is particularly critical in complex food environments, where physical barriers and limited accessibility may restrict effective phage–host contact (Ly-Chatain, 2014; Garcia et al., 2008). Similar variability in adsorption efficiency among lytic bacteriophages has been reported previously, with some phages exhibiting more rapid adsorption dynamics than others during the early stages of host interaction, depending on the phage–host system (Kosznik-Kwaśnicka et al., 2020). Similar adsorption-related limitations affecting phage diffusion and bacterial accessibility in food matrices have also been discussed previously in food biocontrol studies (Guenther et al., 2009).

Following the peak phase, phage titers gradually declined, which may reflect reduced phage stability or environmental influences affecting phage viability over time (Ly-Chatain, 2014). Thermal stability analysis further demonstrated that both phages remained relatively stable at moderate temperatures but gradually lost infectivity at elevated temperatures, consistent with previous reports on food-associated lytic bacteriophages (Sillankorva et al., 2012; Wdowiak et al., 2022). These observations suggest that SW03 and CF03 may be more suitable for application in fresh or minimally processed foods where exposure to high temperatures is limited.

These findings provide a microbiological basis for understanding the observed antibacterial performance under different environmental conditions. Differences in antibacterial performance between SW03 and CF03 may be related to variations in their biological behavior under specific food conditions.

### 4.4 Effect of environmental conditions

Temperature had a clear influence on phage efficacy. Greater reductions were observed at room temperature (21 °C), whereas antibacterial effects were reduced but still detectable under refrigerated conditions (4 °C), consistent with reduced bacterial metabolic activity and limited phage replication at lower temperatures. Similar temperature-dependent effects on bacteriophage performance in food systems have been reported previously (Sillankorva et al., 2012; Guenther et al., 2009; Bigwood et al., 2008).

Detectable antibacterial activity was also observed at the intermediate 4 h time point under both temperature conditions, indicating that phage-mediated bacterial reduction began during the early stages of storage, although the magnitude of reduction increased further after 24 h. Similar observations regarding the influence of storage duration and environmental conditions on phage efficacy have also been discussed in previous food biocontrol studies (Pelyuntha and Vongkamjan, 2023; Taj et al., 2024; Zhao et al., 2023). Nevertheless, the persistence of antibacterial activity under refrigeration conditions suggests that bacteriophages may still contribute to bacterial control during chilled food storage.

The environmental conditions evaluated in the present study provide insight into bacteriophage performance under storage conditions commonly encountered in food systems. The observed differences between refrigerated and room temperature conditions highlight the importance of bacterial physiological activity and environmental factors in determining phage efficacy. In addition, the inclusion of both intermediate (4 h) and extended (24 h) evaluation periods allowed assessment of both early and sustained antibacterial effects under food-related conditions. The use of an optimized MOI further supported effective phage–host interaction under the tested experimental settings.

### 4.5 Comparison with previous food biocontrol studies

To contextualize the findings of the present study, the antibacterial efficacy of SW03 and CF03 was compared with previous bacteriophage biocontrol studies conducted in comparable food matrices and storage conditions (Table 2). Overall, the observed reductions are consistent with previous reports showing that lytic bacteriophages can reduce foodborne bacterial populations in meat and fresh produce. The strongest reductions in the present study were observed in lettuce at 21 °C, whereas reductions in meat matrices were generally lower, supporting the view that food matrix structure, moisture availability, surface characteristics, initial contamination level, and storage temperature can strongly influence phage–host contact and treatment efficacy. Compared with previous studies, the present work is distinguished by its use of high initial bacterial loads, direct comparison across beef, chicken, and lettuce, and evaluation under both refrigerated and room-temperature storage conditions.

**Table 2:** Comparison of the present study with previous bacteriophage biocontrol studies conducted in similar food matrices and storage conditions.

Study	Target pathogen(s)	Food matrix	Storage conditions	Main findings
Present study	*Salmonella enterica*, Escherichia coli O157:H7	Beef, chicken, lettuce	4 and 21 °C	Both phages reduced bacterial populations across all tested food matrices, with the highest reductions observed in lettuce (up to 4.0 and 3.5 log CFU g^−1^ for SW03 and CF03, respectively, after 24 h at 21 °C).
Waturangi et al. (2021)	Enteropathogenic *Escherichia coli* (EPEC)	Chicken meat, lettuce	4°C and room temperature	Bacteriophage treatment reduced EPEC populations in chicken meat and lettuce under both refrigerated and room temperature conditions.
Zhao et al. (2023)	*Salmonella Typhimurium*	Milk, lettuce, raw pork meat, ready-to-eat chicken breast	Various storage temperatures	The phage PS3-1 significantly reduced *Salmonella Typhimurium* populations in lettuce and ready-to-eat chicken breast under different storage conditions.
Pelyuntha and Vongkamjan (2023)	*Salmonella* spp.	Chicken meat	4°C under modified atmosphere packaging	Combined phage-based treatment reduced *Salmonella* populations in chicken meat by up to 4–5 log CFU g^−1^ during refrigerated storage.
Artawinata et al. (2023)	Enterotoxigenic *Escherichia coli* (ETEC), enterohemorrhagic *E. coli* (EHEC)	Chicken meat and lettuce	4 and 28 °C	Bacteriophage treatment reduced ETEC and EHEC populations in chicken meat and lettuce under both 4 and 28 °C conditions, with greater reductions generally observed at 28 °C.

### 4.6 Study limitation

Several limitations should be acknowledged. Experiments were conducted under controlled laboratory conditions using selected food matrices and single phage–host systems, which may not fully represent real food environments. In addition, phage titers were not monitored during the experiments, limiting the ability to distinguish between active replication and passive persistence. Future studies should include monitoring of phage population dynamics during food application experiments to better understand replication behavior under different storage conditions. The use of a single MOI (0.1) in food application experiments also represents a limitation of the present study. Although this MOI was selected based on preliminary optimization experiments, evaluating multiple MOI values may provide a more comprehensive understanding of dose-dependent phage efficacy in food systems. High initial bacterial loads were intentionally used to provide a controlled and reproducible evaluation of bacteriophage activity under stringent conditions. However, such contamination levels are typically higher than those encountered in real food systems. Therefore, the observed reductions should be interpreted as results from a stringent experimental challenge rather than as direct predictions of performance under natural conditions. Future studies should evaluate SW03 and CF03 under lower bacterial loads, naturally contaminated food samples, mixed microbial communities, and industrially relevant handling and storage conditions.

It should also be noted that the crystal violet assay used in this study quantifies total biofilm biomass and does not distinguish between viable and non-viable cells. Therefore, the observed reductions primarily reflect biomass disruption rather than direct measurement of bacterial viability. Furthermore, no structural or imaging-based analyses were performed. The potential development of phage resistance was also not investigated.

### 4.7 Practical implications and future perspectives

From a practical perspective, effective application of bacteriophages in food systems requires optimization of delivery methods and environmental conditions. In this study, phage application was performed via direct surface inoculation, simulating practical approaches such as spraying or dipping. Although both phages retained activity under refrigerated and room temperature conditions, a gradual decline in antibacterial efficacy was observed over time, indicating that extended storage may affect performance. Despite these limitations, the study provides valuable application-relevant insights under controlled yet food-related conditions.

Future studies should incorporate complementary approaches, such as viable cell enumeration or metabolic activity assays, as well as imaging techniques, to provide a more comprehensive evaluation of antibiofilm efficacy.

One important consideration in bacteriophage application is the potential development of bacterial resistance to phages. Similar to antibiotics, bacteria may develop mechanisms to evade phage infection, such as modification or loss of surface receptors. Although resistance development was not evaluated in the present study, it represents an important aspect for future investigation. Strategies such as phage cocktails or combination-based approaches may help reduce the risk of resistance emergence. Further investigation of phage specificity and host–phage interaction dynamics may also help better define the application potential and target range of these bacteriophages in food systems.

### 4.8 Study positioning and implications

Overall, the results demonstrate that bacteriophages SW03 and CF03 effectively reduce high loads of foodborne pathogens across different food matrices under conditions relevant to food storage. The observed variability highlights the importance of both phage biological properties and matrix-specific factors, supporting their potential as biocontrol agents while emphasizing the need for further optimization under practical conditions.

The findings of the present study are consistent with previous research on bacteriophage-based biocontrol in food systems. While earlier mechanistic studies are typically conducted under controlled laboratory conditions, such approaches may not fully capture the complexity of real food environments.

In contrast, the present study adopts an application-oriented approach by evaluating newly isolated bacteriophages (SW03 and CF03) across different food matrices and storage conditions. This allows a more realistic assessment of bacteriophage performance in food systems.

Importantly, the use of high initial bacterial loads in this study provides a stringent and reproducible framework for evaluating bacteriophage efficacy under challenging conditions. This approach strengthens the assessment of phage performance and enhances the practical relevance of the findings.

Therefore, the current study contributes to bridging the gap between controlled experimental research and the practical application of bacteriophage-based biocontrol strategies in food systems.

## 5 Conclusion

This study demonstrates that lytic bacteriophages SW03 and CF03 effectively reduce high loads of their corresponding bacterial targets, *Salmonella enterica* and Escherichia coli O157:H7, respectively, in both *in vitro* systems and food matrices under refrigerated and room-temperature storage conditions. Among the two phages, SW03 generally showed higher antibacterial activity and greater reduction of preformed biofilm biomass under the tested conditions.

Phage efficacy was influenced by environmental factors, particularly temperature and food matrix type, with the highest antibacterial reductions observed in lettuce samples at 21 °C. Detectable antibacterial activity was also maintained under refrigerated conditions, although reductions were comparatively lower.

Taken together, this study provides application-oriented insight into bacteriophage performance under food-related and high-inoculum challenge conditions and supports the potential use of the two isolated bacteriophages as biocontrol agents in food safety applications.

Further studies are required to optimize application strategies and evaluate their effectiveness under industrial and large-scale food processing conditions.

## Data Availability

The genome sequence data generated in this study are available in GenBank under accession numbers PX705374.1 for phage SW03 and PX705375.1 for phage CF03. All other data supporting the findings of this study are included in the article and/or supplementary material.
